# A new species of *Dianthus* (Caryophyllaceae) from Antalya, South Anatolia, Turkey

**DOI:** 10.3897/phytokeys.63.8033

**Published:** 2016-04-26

**Authors:** İsmail Gökhan Deniz, Candan Aykurt, İlker Genç, Ahmet Aksoy

**Affiliations:** 1 Department of Biology Education, Faculty of Education, Akdeniz University, Antalya, Turkey; 2 Department of Biology, Faculty of Science, Akdeniz University, Antalya, Turkey; 3 Istanbul University, Faculty of Pharmacy, Department of Pharmaceutical Botany, Istanbul, Turkey

**Keywords:** Dianthus, new species, taxonomy, Turkey

## Abstract

*Dianthus multiflorus* from Gazipaşa (Antalya), south Anatolia (Turkey), is described as a new annual species with verrucose calyx. The morphological differences from the species within the same group with *Dianthus multiflorus*, which are *Dianthus aydogdui*, *Dianthus cyri* and *Dianthus tripunctatus*, are discussed. The International Union for Conservation of Nature (IUCN) threat category and observations on the ecology of the populations are noted. The karyology and seed micromorphology of *Dianthus multiflorus* and *Dianthus tripunctatus* were examined by light microscopy and scanning electron microscopy.

## Introduction

Amongst all its neighbouring countries, Turkey is the richest in terms of plant taxa, being home to 9996 plant species (11707 taxa) (Güner et al. 2012). The floristic richness of the country is partially due to the high number of endemic and rare species present. The Mediterranean region is one of the important centers of endemism in Turkey, and Antalya is the richest province of Turkey in terms of plant diversity, hosting 773 of the country’s endemic species. Amongst these endemic species, about 244 are best described as locally endemic, being found only in Antalya (Deniz and Aykurt 2014).

After *Silene* L., *Dianthus* L. is the second largest genus of Caryophyllaceae. This genus, containing approximately 300 species, is mainly distributed in the Mediterranean region of Europe and Asia (Reeve 1967; Bittrich 1993). The most comprehensive study on *Dianthus* species in the Flora of Turkey and East Aegean Islands was carried out by Reeve (1967) wherein 67 species were recorded. Since that date, new species and records have been added and the total number of *Dianthus* species recorded in Turkey has increased to 81 (Shishkin 1985, Davis et al. 1988, Gemici and Leblebici 1995, Güner 2000, Menemen and Hamzaoğlu 2000, Aytaç and Duman 2004, Özhatay and Kültür 2006, Vural 2008, Yılmaz et al. 2011, İlçim et al. 2013, Hamzaoğlu 2012, Hamzaoğlu and Koç 2015, Hamzaoğlu et al. 2014, 2015a, 2015b, 2015c). Among the annual *Dianthus* species recognized by Reeves (1967), there are only two species (*Dianthus cyri* Fisch. & C.A.Mey. and *Dianthus tripunctatus* Sm.) that have a verrucose calyx.

*Dianthus aydogdui* Menemen & Hamzaoğlu, which resembles *Dianthus cyri* and *Dianthus tripunctatus*, has been described from Salt Lake (Central Anatolia) province by Menemen and Hamzaoğlu (2000). The new species described here, *Dianthus multiflorus* Deniz & Aykurt, was collected from Gazipaşa province in Antalya. It is distributed on stony sliding slopes and serpentine soils in clearings of *Pinus brutia* forest. *Dianthus multiflorus* is included in the same group along with *Dianthus aydogdui*, *Dianthus cyri* and *Dianthus tripunctatus*. *Dianthus multiflorus* shows distinct differences from these species by its habit, number of flowers on the stem, calyx and petal features.

## Methods

### Plant samples and morphological studies

*Dianthus multiflorus* specimens were collected from Gazipaşa province (Antalya) during field studies within the scope of the project “EXPO 2016 Endemic and Rare Flowers of Antalya”. New species and its morphologically most similar species, *Dianthus tripunctatus* were observed during field studies and their morphological characteristics were recorded both in the field and in the laboratory. Specimens collected were comprehensively evaluated by the use of the literature (Velenovsky 1891; Post 1932; Tutin 1964; Reeve 1967; Shishkin 1985; Strid 1986; Rechinger 1988) and the specimens present in GAZI, ISTE, and Akdeniz University herbariums. The overall morphology of the new species was examined with stereo-binocular microscope.

The seed micromorphology of *Dianthus multiflorus* and *Dianthus tripunctatus* was investigated using scanning electron microscopy (SEM) techniques. Seeds were attached to SEM stubs, coated with gold conjugate following the manufacturer specifications and examined with a Zeiss LEO-1430 scanning electron microscope.

### Karyological studies

Chromosome number and karyological features of the *Dianthus multiflorus* and *Dianthus tripunctatus* were determined from plant material collected from Antalya. All karyological observations were carried out on root tips. Root-tip meristems were provided from seeds by germinating them on wet filter paper in petri dishes at the temperature of 23 °C. Firstly, root tips were pretreated for 24 h in a-monobromonaphthalene at 4 °C, fixed in 3:1 absolute alcohol-glacial acetic acid. Root tips were then hydrolyzed with 1 N HCL for 13 min. at 60 °C, stained in Feulgen solution, and squashed in acetoorcein. For karyotype analysis, the photographs were taken using OLYMPUS BX53 microscope with camera Kameram 12 CCD attachment. Chromosome counts in mitosis metaphase and karyotype analyses were obtained based on five root tips, five metaphase cells for each individual. Measurements of somatic chromosomes were made with the program KAMERAM, they were calculated with formula of the relative variation in chromosome length CV_CL_ (Paszko 2006), mean centromeric asymmetry (M_CA_) according to Peruzzi and Eroğlu (2013) and chromosome total haploid length (THL) (Peruzzi et al. 2009). Chromosomes were classified to the nomenclature following Levan et al. (1964) and asymmetry types following Stebbins (1971).

## Results

With the recognition of *Dianthus multiflorus* as a new species, there are now four annual species of *Dianthus*, (*Dianthus multiflorus*, *Dianthus tripunctatus*, *Dianthus cyri* and *Dianthus aydogdui)* that can be separated from other annual *Dianthus* species on the basis of their verrucose calyxes in Turkey. A detailed comparision of the morphological and ecological features of these species is shown in the Table 1.

**Table 1.** Comparison of diagnostic morphological characters of *Dianthus multiflorus* with its close relatives.

Characters	*Dianthus multiflorus*	*Dianthus tripunctatus*	*Dianthus cyri*	*Dianthus aydogdui*
**Plant size (cm)**	20–80	20–50	12–40	3–15
**Flowering stem**	Divaricately branched	Many branched	Many branched	Single stemmed
**Epicalyx scales length**	Almost equaling or shorter than calyx tube	Equaling calyx tube	Equaling or longer than calyx tube	Shorter than calyx tube
**Epicalyx scales mucro length (mm)**	3–3.5	3–8	7–12	1–3
**Pedicels length (mm)**	0.5–30	40–50	15–35	5–15
**Number of flowers**	Numerous (30–) 80–250 (–300)	4–15 (–40)	5–22	1–4
**Calyx length (mm)**	12–13	18–20	11–15	8–10
**Calyx tube**	Distinctly 35–40 nerved	Nervose-striate	Enervate	Nervose
**Apex of calyx teeth**	Acuminate	Acuminate	Aristate	Acute sometimes mucronate
**Petal limb color**	White with purple venation	Pink	Pink	Pink
**Margin of petal limb**	Emerginate with shallowly sinuate lobes	Dentate	Dentate	Dentate
**Ecology**	Sliding slopes and serpentine soils	Cliffs, road sides	Deep alluvial soils	Salty soils
**Altitude**	1000–1150 m	1–120 m	1200 m	950 m

### Dianthus multiflorus

Taxon classificationPlantaeCaryophyllalesCaryophyllaceae

Deniz & Aykurt sp. nov.

urn:lsid:ipni.org:names:77154520-1

**Figure 1.**
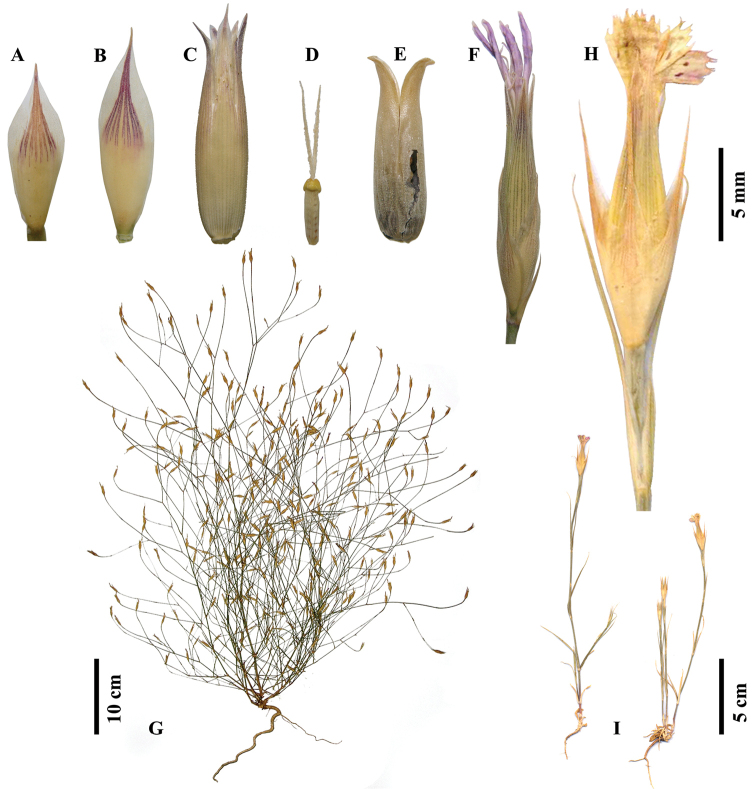
Habit and flower photographs of *Dianthus multiflorus* (**A–G** from holotype, *Deniz 6195*) and *Dianthus tripunctatus* (**H–I** from ISTE 74221). (**A** Outer epicalyx segment **B** Inner epicalyx segment **C** Calyx **D** Pistil **E** Capsule **F, H** Flower **G, I** Habit).

**Figure 2.**
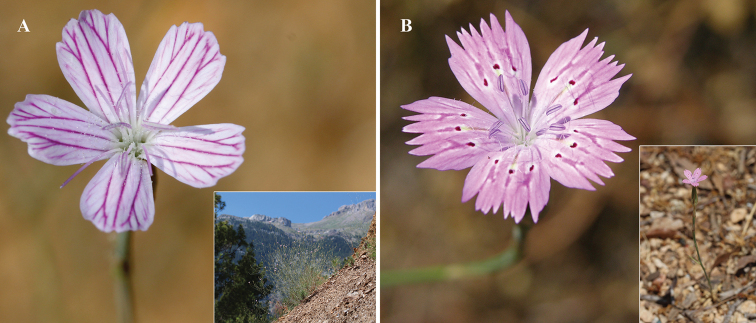
Field photographs of *Dianthus multiflorus* (**A**) and *Dianthus tripunctatus* (**B**).

**Figure 3.**
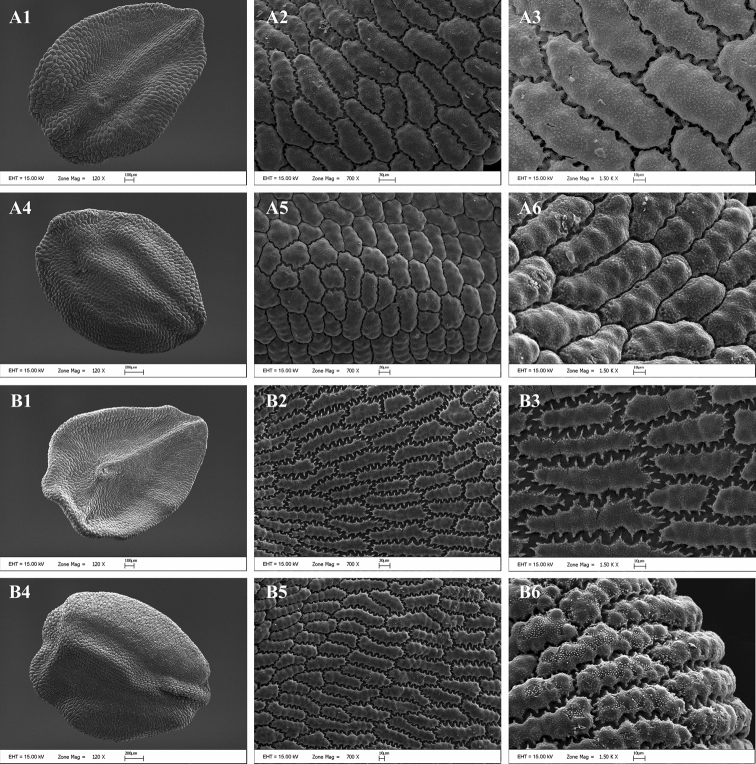
SEM photographs of the seed coat. **A** *Dianthus multiflorus* **B** *Dianthus tripunctatus*. 1–3: Ventral surface. 4–6: Dorsal surface. (Scale bars 200 μm for A4 and B4; 100 μm for A1 and B1; 30 μm for A2; 20 μm for A5 and B2; 10 μm for A3, A6, B3, B5 and B6).

**Figure 4.**
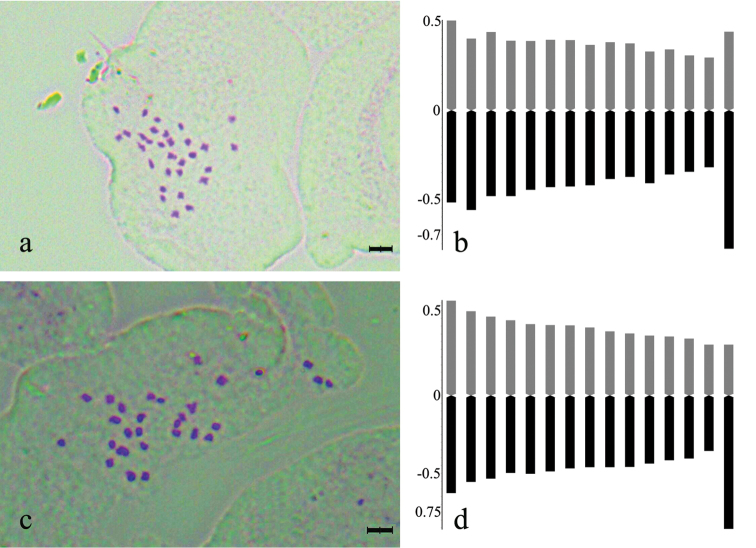
Somatic chromosomes and ideogram of *Dianthus multiflorus* (**a–b**) and *Dianthus tripunctatus* (**c–d**). (Scale bars 2 μm).

Figs 1 , 2 , 3 , 4 , Table 1 , 2

#### Diagnosis.

*Dianthus multiflorus* is distinguished from related species by having flowers numerous [(30–)80–250(–300)], pedicels 0.5–30 mm, calyx 12–13 mm, petals white, and petal limb margins shallowly sinuate.

#### Type.

TURKEY. Antalya: Gazipaşa, from Akoluk Village to Akkaya Hill of Taşeli Plateau, c. 3. km, stony sliding slopes and serpentine soils in clearings of *Pinus brutia* forest, 1075 m a.s.l., 05 July 2015, *İ.G. Deniz*, C. *Aykurt*, *6195* (holotype: Akdeniz University Herbarium 3823).

Annual, many-stemmed, divaricately branched herbs. Stem erect to ascending, fragile, slender, 20–80 cm, branching from the base, glabrous or minutely scabridulous especially toward base, usually purplish at base, many-flowered (30–)80–250(–300). Basal leaves linear-lanceolate to lanceolate-spatulate, 25–35 × 2.5–4.5 mm, with scabridulous edges especially near base, obtuse at apex. Cauline leaves linear-narrowly triangular to linear-lanceolate, gradually smaller upwards, flattened, distinctly nervous, glabrous, with scabridulous margin and narrowly membranous toward base, acute to acuminate at apex, their sheaths shorter than the internodes; lower cauline leaves linear-lanceolate, 20–50 × 2–2.2 mm, longer or shorter than internodes, swollen and usually purplish at base; upper cauline leaves linear to linear-narrowly triangular, 4–20 × 0.5–1.5 mm, slightly swollen at base. Inflorescence dichotomously branched; flowers almost always solitary, occasionally two or three flowers borne on the same nod; branches usually minutely scabridulous; pedicels 1–30 mm (sometimes very short, to 0.5 mm). Epicalyx scales 4, almost equaling or shorter than calyx tube, cartilaginous, straw-colored, markedly 8-nerved toward apex, glabrous, verrucose at middle and below surfaces, scabridulous at apex and on scarious margins; scarious margins terminating at or under apex; outer epicalyx segments obovate, 8–9 × 3–3.5 mm, with acuminate tip (ca. 3 mm), scarious margins up to 0.75 mm broad; inner epicalyx segments obovate, 11–12 × 4–4.5 mm, with acuminate tip (ca. 3.5 mm), scarious margins up to 0.8 mm broad. Calyx cylindric-lanceolate, verrucose, distinctly 35–40-veined, usually purplish at upper 2/3 part, 12–13 × 2.8–3 mm; teeth triangular, 4.5–5.5 mm long, 7–8–veined, with narrowly scarious and scabridulous margins. Petals white, 20 mm; limb narrowly obovate, 6–7 × 3 mm, emarginate with shallowly sinuate lobes, completely exerted from calyx, unspotted, barbulate, with 3 main purplish vein; claw 12–13 × ca. 1 mm. Anthers 3.6 mm long; filaments 7 mm long. Ovary 3.5 mm long; style 5 mm long. Capsule cylindrical, included in calyx, 10 × 3 mm. Seeds ovate to elliptic, 1.9–2.5 × 1.07–1.7 mm, black, minutely cuspidate at apex, granular, covered by irregularly polygonal or rectangular cells; anticlinal walls represented by shallow and wide grooves, with U-like undulations; the periclinal walls distinctly papillose. The cells of ventral surface 75–145 × 33.2–59.4 μm, more elongated and bigger than the cells of dorsal surface (31.4–86 × 25–45 μm).

#### Distribution, habitat and ecology.

*Dianthus multiflorus* is known only from the type locality, between Akoluk Village to Taşeli Plateau, where it grows at altitude of 1000–1150 m on sliding slopes and serpentine soils in clearings of *Pinus brutia* Ten. forest. Within this area, the new taxon is associated with plants such as: *Pinus brutia* var. *brutia*, *Quercus coccifera* L., *Helichrysum arenarium* Moench subsp. *aucheri* (Boiss.) P.H.Davis & Kupicha, *Carduus rechingerianus* Kazmi, *Centaurea urvillei* DC. subsp. *urvillei*, *Teucrium lamiifolium* d’Urv. subsp. *lamiifolium*, *Thymus cilicicus* Boiss. & Balansa. *Ballota saxatilis* Sieber ex C.Presl subsp. *saxatilis*.

#### Phenology.

*Dianthus multiflorus* was observed flowering in June and July, and mature fruits are produced in July to middle of August.

#### Etymology.

The species epithet is derived from its abundant flowers representing one of the main characters that distinguishes it from other similar species.

#### Proposed conservation status.

*Dianthus multiflorus* is included in the Critically Endangered category according to IUCN criteria ver. 11 (IUCN 2014). The species was determined at only a single location and the extent of occurrence (EOO) value of the species was determined to be 7 km^2^ taking into account location of occupancy and the area contained within the shortest continuous imaginary boundary. Additionally, the area of occupancy (AOO) value in this area was calculated as 4 km^2^ [CR B1ab(i)+CR B2b(ii)].

#### Seed testa micro-morphology.

A detailed comparison of seed micromorphology based on SEM analysis was made (Table 2). The seeds of *Dianthus multiflorus* and *Dianthus tripunctatus* are black, minutely cuspidate at apex, granular, and have anticlinal walls represented by shallow and wide grooves, with U-like undulations, while the periclinal walls are distinctly papillose. The seeds of *Dianthus multiflorus* are covered by irregularly polygonal or rectangular cells, and the cells of ventral surface are more elongated and larger, conversely the seeds of *Dianthus tripunctatus* are covered by irregularly rectangular cells and the cell size of ventral and dorsal surfaces are similar (Fig. 3).

**Table 2.** Comparison of seed micromorphological and karyological characteristics of *Dianthus multiflorus* and *Dianthus tripunctatus*.

	**Characters**	***Dianthus multiflorus***	***Dianthus tripunctatus***
Seed micromorphology	Seed size (mm)	1.9–(2.16)–2.5 × 1.07–(1.46)–1.07	1.9–(2.07)– 2.23 × 1.4–(1.62)–1.87
	Cell shape of seed coat	Polygonal or rectangular	Rectangular
	Cell size of ventral surface	75–145 × 33.2–59.4 μm	(60–)97.4–127.3 × 18.2–26
	Cell size of dorsal surface	31.4–86 × 25–45 μm	57–140 × 18.6–32.9
	Cell size of ventral surface according to dorsal surfaces	More elongated and bigger	Similar
Karyology	SC LC THL M_CA_ CV_CL_ Stebbins symetry	0,54 μm 1.24 μm 12.21 μm 6.26 19.93 3B	0.77 μm 1.21 μm 14.09 μm 6.42 12.57 3A

#### Karyology.

The chromosome number of *Dianthus multiflorus* and *Dianthus tripunctatus* is 2*n* = 30 (Fig. 4). The shortest chromosome length for *Dianthus multiflorus* is 0.54 μm, the longest is 1.24 μm, and total haploid chromosome length (THL) is 12.21 μm. The karyotype formula of *Dianthus multiflorus* consists of 28 median pairs and 2 submedian pairs. As for karyotype asymmetry, its karyotype was classified according to the symmetry classes of Stebbins (1971) as 3B. Intrachromosomal asymmetry (M_CA_) is 6.26 and the interchromosomal asymmetry index (CV_CL_) is 19.93. Our study showed that the shortest chromosome length for *Dianthus tripunctatus* is 0.77 μm, the longest is 1.21 μm, and total haploid chromosome length (THL) is 14.09 μm. The karyotype formula of this species consists of 28 median pairs and 2 submedian pairs. As for karyotype asymmetry, the karyotype of this species is classified according to the symmetry classes of Stebbins (1971) as 3A. Intrachromosomal asymmetry (M_CA_) is 6.42 and the interchromosomal asymmetry index (CV_CL_) is 12.57. The karyogram is given in Figure 4 and ideogram was drawn based on the centromeric index (Fig. 4).

According to Stebbin’s (1971) classification, the karyotypes of *Dianthus multiflorus* belong to type 3B, whereas the karyotypes *Dianthus tripunctatus* belong to type 3A. The asymmetry indices also reveal some small differences between the two species. Nevertheless, according to interchromosomal asymmetry index (CV_CL_), *Dianthus multiflorus* is more asymmetric than *Dianthus tripunctatus*. The total haploid chromosome length (THL) in *Dianthus multiflorus* is 12.21 μm and that of *Dianthus tripunctatus* is 14.09 μm (Table 2).

##### Diagnostic key to the annual *Dianthus* species with verrucose calyx in Turkey

**Table:** 

1	Stem unbranched, 3–15 cm; calyx 10 mm or shorter	***Dianthus aydogdui***
–	Stem many branched, 12–80 cm; calyx more than 10 mm	**2**
2	Stem bearing (30–)80–250(–300) flowers; petal limb white with purple venation, shallowly sinuate at margins	***Dianthus multiflorus***
–	Stem bearing 4–15(–40) flowers; petal limb pink, dentate at margins	**3**
3	Calyx tube nervose-striate; base of epicalyx segments adpressed to the calyx, their membranous margins conspicuous, 2 mm wide	***Dianthus tripunctatus***
–	Calyx tube enervate; base of epicalyx segments spreading, their membranous margins inconspicuous, not more than 0.5 mm wide	***Dianthus cyri***

## Discussion

Of the annual *Dianthus* species that occur in Turkey, there are only four species that have verrucose calyxes; *Dianthus aydogdui*, *Dianthus cyri*, *Dianthus multiflorus* and *Dianthus tripunctatus*. *Dianthus aydogdui* was recently described from Salt Lake province (Menemen and Hamzaoglu 2000), and this species is distinct from *Dianthus tripunctatus* and *Dianthus Cyri* due to its short and single stems, and shorter epicalyx scales in relation to its calyx tube. *Dianthus multiflorus* is also closely related to *Dianthus tripunctatus* and *Dianthus cyri*. In particular, the habit and floral characteristics of *Dianthus multiflorus* are quite different from other species. Its divaricately branched stems bear numerous flowers unlike those of *Dianthus tripunctatus* and *Dianthus cyri*. Contrary to the pink and dentate petal-limbs of *Dianthus tripunctatus* and *Dianthus cyri*, *Dianthus multiflorus* has white petals with distinct dark purplish venation and emarginate with shallowly sinuate margins. *Dianthus multiflorus* shows more resemblance to *Dianthus tripunctatus* than *Dianthus cyri* by the length of its epicalyx scales and the features of calyxes. The calyx tubes of *Dianthus multiflorus* and *Dianthus tripunctatus* are nervose-striate whereas those of *Dianthus cyri* are enervose. However, the calyx length of *Dianthus multiflorus* is shorter than that of *Dianthus tripunctatus*. Further, the epicalyx scales are shorter to almost equaling in the calyx tube in *Dianthus multiflorus* whereas they are equaling in *Dianthus tripunctatus* and shorter in *Dianthus cyri*. *Dianthus multiflorus* and *Dianthus tripunctatus* have acuminate calyx teeth compared to their being aristate in *Dianthus cyri*.

Besides morphological characteristics, both seed micromorphological and karyological features of *Dianthus multiflorus* and *Dianthus tripunctatus* were also identified within the present study. According to the results of the seed micromorphological studies, the main difference between the seeds of *Dianthus multiflorus* and *Dianthus tripunctatus* is the shape and size of the coat cells. According to the results of the karyological studies, there are some karyomorphological differences between the two species. According to Levin (2002), the correlation between THL and 1C values within and between species in related genera, THL is considered a good proxy for genome size. On this basis, Peruzzi and Altınordu (2014) proposed a standardized method, taking into account six quantitative parameters, in order to establish relationships among taxa. THL is one of these parameters and the total haploid chromosome length (THL) in *Dianthus multiflorus* was comparatively lower than that of *Dianthus tripunctatus*.

## Conclusion

The most important reason for the high endemism values in the Antalya and Mediterranean regions of Turkey is the sudden climatic and topographic differences. The Taşeli Plateau, which is one of the endemism centers of the eastern part of the Antalya province, is at an altitude of approximately 2200 meters and is just 20 km from the Mediterranean Sea. The lower slopes of the region are characterized by a typical Mediterranean climate and vegetation types, but the climatic conditions are continental at higher altitudes. The distribution area of *Dianthus multiflorus* is located in these climatic and topographic transition regions, and discovery of the new species contributes to a better understanding the richness of the Turkish Flora. With this study, the total number of species belonging to the genus *Dianthus* has risen to 82, the study provides material and data to aid further research on *Dianthus*, an important member of the Caryophyllaceae.

**Specimens Examined. *Dianthus aydogdui* Menemen & Hamzaoğlu**–TURKEY. Aksaray: Salt lake province, The north way from Ulukışla to Salt Lake, 950 m, 24 June 1999, *E. Hamzaoğlu & M. Aydoğdu 2432* (isotype GAZI!); ***Dianthus cyri* Fisch. & C.A.Mey.**–TURKEY: 06 July 1970, *F. Sorger 70–43–1* (E!) http://data.rbge.org.uk/herb/E00475274; UNITED ARAB EMIRATES. Fujeirah Coast–Lulayyah: Open fields in shelttered coastal plantation, 26 February 1986, *R.A. Western 881* (E!) http://data.rbge.org.uk/herb/E00181825; ***Dianthus tripunctatus* Sm.**–TURKEY. Antalya: Karayolları beach, 40 m, 15.6.1983, *H. & G. Çakırer s.n.* (ISTE 50905!); Antalya: Ulaş Highway recreation park, 20 km from İncekum to Alanya, s.l., 19 June 1983, *H. & G. Çakırer s.n.* (ISTE 51106!); Antalya: Kemer, Çıralı, under *Pinus brutia*, 10 m, 5 May 2013, *R. Süleyman Göktürk 7621* (Akdeniz University Herbarium 1779!); İzmir: 30 May 1960, *N. Öktem 56* (ISTE 6218!); Muğla: Ortaca, between Dalyan and Tepe, 10–20 m, roadsides, 18 June 1991, *A. Güner 9501* (GAZI!).

## Supplementary Material

XML Treatment for Dianthus multiflorus
